# Enhancing Bromelain Recovery from Pineapple By-Products: A Sustainable Approach for Value Addition and Waste Reduction

**DOI:** 10.3390/foods13040589

**Published:** 2024-02-15

**Authors:** Peter G. Chiarelli, Bismarck Martinez, Takashi Nakamura, Kevin Mis Solval

**Affiliations:** 1 Department of Food Science and Technology, The University of Georgia, Griffin, GA 30223, USA; 2 Research and Development Department, Del Monte Fresh Produce NA, 241 Sevilla, Miami, FL 33134, USA

**Keywords:** pineapple by-products, bromelain, dialysis, enzymatic activity, upcycling

## Abstract

Pineapple by-products are good sources of bromelain, a complex enzyme with commercial applications. This study evaluated the feasibility of producing bromelain powders from pineapple waste using an organic solvent-free approach. Pineapple by-products (from var. MD2), including cores, peels, crowns, stems, and basal stems, were homogenized with deionized water, and the pH of the mixture was adjusted to 4.5 and 9 (isoelectric points of fruit bromelain and stem bromelain), clarified, ultra-filtered, and freeze-dried to produce bromelain powders. The enzymatic activity of the bromelain powders was measured using the gelatin and casein digestion methods. The bromelain powders from the crowns did not show significant enzymatic activity (*p* < 0.05). Meanwhile, bromelain powders produced from the cores and peels had an enzymatic activity of 694 gelatin digesting units (GDU)/g and 124 casein digesting units (CDU)/mg, and 1179 GDU/g and 217 CDU/mg, respectively. Bromelain powders from the basal stems showed the highest enzymatic activity (2909 GDU/g and 717 CDU/mg). Increasing the pH of the liquid mixture before the purification and freeze drying significantly (*p* < 0.05) reduced the enzymatic activity of the bromelain powders. Using a practical and organic solvent-free approach, this study demonstrates the feasibility of producing bromelain powders with high enzymatic activity from pineapple waste.

## 1. Introduction

Pineapple (*Ananas comosus* L. Merr.) is a tropical fruit that ranks third in popularity worldwide, after bananas and citrus fruits. Furthermore, pineapples are recognized for their unique taste and nutrients with health benefits, including vitamins C, A, B_2_, B_3_, B_6_, and bromelain [1]. This makes pineapples a smart choice for health-conscious consumers looking to improve their diet.

Surprisingly, 55–70% of the pineapple is typically considered waste (peels, cores, crowns, and trimmings), with only about 30–45% being pulp [2]. Sadly, this pineapple waste often ends up in landfills or is used as fertilizer or animal feed [3]. Current global sustainability and food waste reduction efforts are creating exciting opportunities for the global pineapple industry to turn this waste into value-added products. Several initiatives have recently emerged to create high-value products such as citric and lactic acids, ethanol, xylooligosaccharides, and bromelain [4,5,6]. 

Bromelain is a term used to describe a group of enzymes found in the tissues of the plant family Bromeliaceae. It is a versatile endopeptidase widely used in food, cosmetics, and chemical and pharmaceutical applications [7]. In the food and animal industries, it plays a critical role in meat tenderizing, beer clarification, baking, and producing valuable protein hydrolysates [8]. Interestingly, bromelain can be found not only in the pulp, but also in the core, stem, and peels of pineapples, and has a molecular weight between 23 and 36 kDa [7]. 

According to De Lencastre Novaes et al. [9], bromelain is a pretty complex mixture of endopeptidases and non-protease elements, including phosphatases, glycosidases, peroxidases, cellulases, glycoproteins, ribonucleases, and carbohydrates. Recent research using whole-genome sequencing of the MD2 pineapple variety (also known as “sweet golden” or “golden”) has identified 14 genes associated with bromelain production, suggesting that these 14 proteases may make up the bromelain activity in pineapples [10]. Interestingly, bromelain is soluble in water but not in organic solvents. Also, its isoelectric point (pI) varies, with a pI of 4.6 for bromelain from pulp and cores and a pI of 9.5 for bromelain from crowns, peels, and stems [11]. 

Researchers have recently suggested that bromelain can boast numerous health benefits, including prophylactic, antibiotic, and anti-thrombotic effects, among others [12,13]. Therefore, bromelain (with high enzymatic activity) may be used to aid digestion and as a preventive measure for conditions like rheumatoid arthritis, oral inflammation, and diabetic ulcers [12,13].

Although commercially available bromelain-based products are primarily extracted from pineapple stems, research has shown that significant amounts of bromelain can also be found in the core, peel, and crown of pineapples [14]. 

Several methods have been used to extract bromelain from pineapple by-products, including reverse micellar technology, solvent extraction, salt precipitation, and ultrafiltration [2,7,15,16]. Additionally, chemical precipitation, filtration, and freeze-drying can be used to extract bromelain with some success [15]. Unfortunately, bromelain extraction using organic solvents (such as acetone, hexane, ethyl acetate, and ethanol) may also extract unwanted compounds that interfere with its enzymatic activity. Moreover, removing the solvent before use in some applications can be cumbersome and costly [17]. Therefore, there is a current need to develop practical methods for bromelain extraction that do not require chemical solvents. 

Different methods have been used to measure the enzymatic activity of extracted bromelain, such as the casein or gelatin digestion methods. The extraction process and the quantification method mean that the quantification of the bromelain activity in these samples can be complex, and unfortunately, there is a lack of consensus regarding the best method to report the enzymatic activity of bromelain. These facts make comparing extraction techniques and commercially available dietary supplements challenging. 

To tackle the high costs associated with the production of bromelain-based products, we have hypothesized that high-enzymatic-activity bromelain can be extracted from pineapple by-products using an organic solvent-free approach. The objective of this research project was to evaluate the feasibility of processing bromelain powders from pineapple wastes utilizing ultrafiltration and freeze-drying processes, as well as and the characterization of the obtained product using the casein and gelatin digestion methods. This approach can potentially produce high-quality bromelain suitable for use as a functional ingredient in food or dietary supplements. 

## 2. Materials and Methods

### 2.1. Materials

#### 2.1.1. Pineapple By-Products

##### Cores, Peels, and Crowns

Pineapple by-products, including cores, peels, and crowns, were provided by Del Monte Fresh Produce NA. Furthermore, all by-products (from the pineapple variety MD-2) were collected and shipped from a US commercial fresh-cut processing facility. By-products were derived from high-quality pineapples with less than 10% defects and 14° brix (Bx) in the flesh. The by-products were shipped in refrigerated conditions to the University of Georgia (UGA)—Food Product Innovation and Commercialization Center (FoodPIC) facilities in Griffin, GA, USA. All samples were kept at 4 °C until needed for experiments, and the samples were processed within the first week of arrival.

##### Stems and Basal Stems

Fresh pineapple and basal stems were obtained from a pineapple plantation located in Costa Rica. First, an import permit was obtained from the U.S. Department of Agriculture (USDA)—Animal and Plant Health Inspection Services (APHIS). Then, the fresh samples were shipped to the UGA FoodPIC facilities. Upon arrival, the samples were stored at −20 °C until needed for experiments and analysis.

##### Chemicals

We used sodium hydroxide anhydrous pellets (>97% purity, 795429, Sigma-Aldrich, St. Louis, MO, USA); gelatin powder from bovine skin (Type B, G9391, Sigma-Aldrich, St. Louis, MO, USA); sodium chloride (>99% purity, S640, Fisher Scientific, Hampton, NH, USA); anhydrous disodium phosphate (1.06585, Sigma-Aldrich, St. Louis, MO, USA); casein, Hammarsten bovine (E0789, Sigma-Aldrich, St. Louis, MO, USA); trichloroacetic acid (>99% purity, T6399, Sigma-Aldrich, St. Louis, MO, USA); sodium acetate (>99% purity, S2889, Sigma-Aldrich, St. Louis, MO, USA); L-cysteine hydrochloride (>98% purity, C1276, Sigma-Aldrich, St. Louis, MO, USA); L-tyrosine (1.08371, Sigma-Aldrich, St. Louis, MO, USA); ethylenediaminetetra-acetic acid (EDTA) (>99.4% purity, E9884, Sigma-Aldrich, St. Louis, MO, USA); hydrogen peroxide (HP) (30% purity, Sigma-Aldrich, St. Louis, MO, USA); acetic acid (>99.7% purity, Fisher Scientific, Hampton, NH, USA); 6 N hydrochloric acid (72033, Honeywell, Charlotte, NC, USA); and 37% formaldehyde (FA) (CAS No. 50-00-0, Carolina Biological Supply Company, Burlington, NC, USA).

### 2.2. Experimental Approach

This research project was divided into three phases.

#### 2.2.1. Phase I—Feasibility Studies

**Figure 1:**
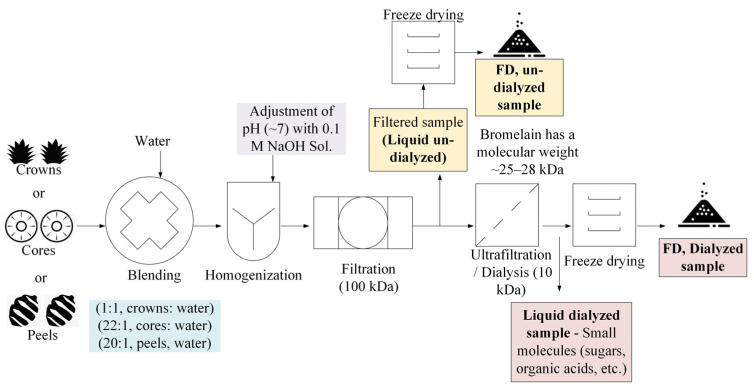
Overview of producing freeze-dried (FD) bromelain powders from pineapple cores, peels, and crowns.

**Figure 2:**
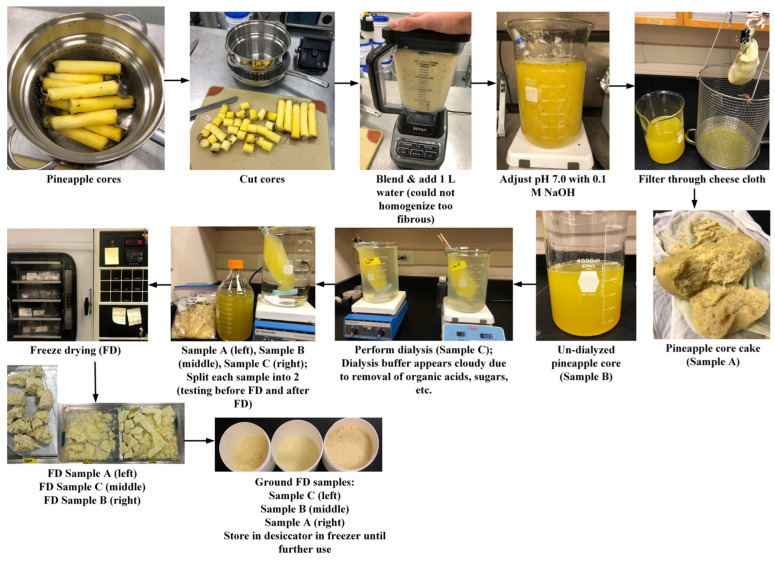
Production of freeze-dried (FD) bromelain powders from pineapple cores and peels.

**Figure 3:**
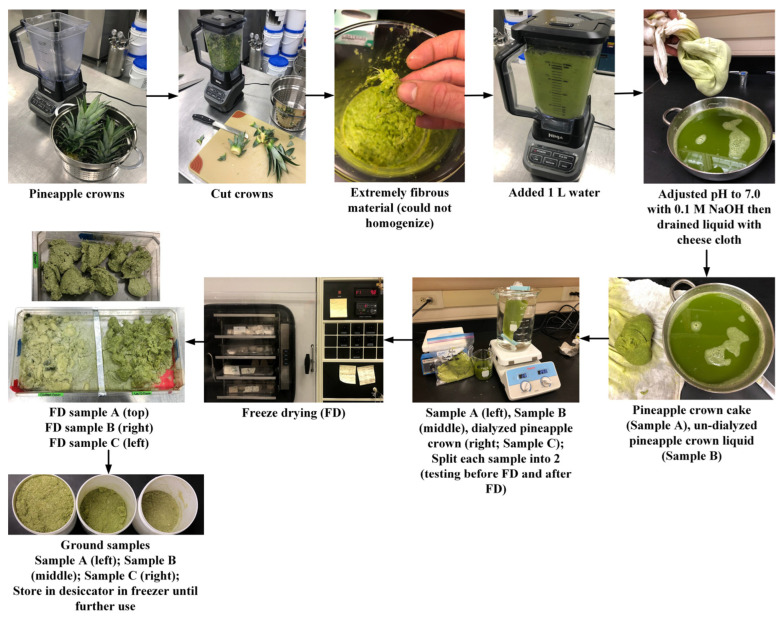
Production of freeze-dried (FD) bromelain powders from pineapple crowns.

In phase I, the feasibility of producing freeze-dried (FD) bromelain powders from pineapple cores, peels, and crowns was evaluated. In short, pineapple by-products were chopped and blended with deionized water (DIW) at ratios of 1:1 (crowns/DIW), 22:1 (cores/DIW), and 20:1 (peels/DIW). Then, the mixtures were homogenized, and the pH was adjusted to 7 with a 0.1 M NaOH solution. The pH adjustment was carried out to precipitate most of the bromelain present in the pineapple by-products, considering that fruit bromelain has a pI of 4.5; meanwhile, stem bromelain has a pI of 9.5 [18]. The pI is the pH at which a protein does not have a net charge and its solubility is minimal. Afterward, the mixtures were filtrated (producing cake and liquid). Then, the liquid was ultrafiltered (dialyzed) using regenerated cellulose dialysis tubes (cut 25–30 mm in length) (MWCO: 6–8 kDa, Spectra/Por^®^ 1 Dialysis Membrane, Spectrum Laboratories, Inc., Rancho Dominguez, CA) at room temperature for ~12 h. This process yielded two samples (un-dialyzed liquid and dialyzed liquid). The molecular weight of pineapple bromelain is between 25 and 32 kDa [7]. Then, the samples were FD and ground to produce FD bromelain powders (Figure 1). This approach was created based on the team’s previous experience and an extensive literature review. The resultant bromelain powders obtained from the cores and peels (Figure 2) and the crowns (Figure 3) were evaluated for crude protein content and enzymatic activity (using the gelatin digestion method described in Section 2.3.3).

#### 2.2.2. Phase II—Improved Bromelain Extraction

In phase II, the production of FD bromelain powders from cores and peels was further improved. Moreover, the enzymatic activity of commercially available bromelain products was determined and compared against those of the resultant FD bromelain powders (using the casein and gelatin digestion methods described below).

##### Modified Production of Bromelain Powders from Pineapple Cores

**Figure 4:**
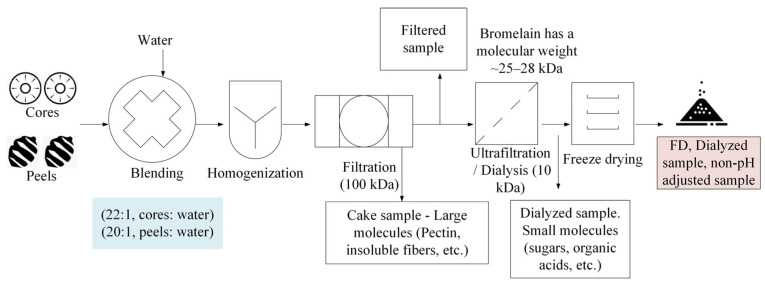
Production of freeze-dried (FD) bromelain powders from pineapple cores and peels without pH adjustment.

Approximately 1 kg of pineapple cores was blended and homogenized with DIW at a ratio of 22:1 (*w*/*w*). Then, the homogeneous mixture was filtered, dialyzed, and FD to produce FD bromelain powders from pineapple cores (Figure 4). Alternatively, FD bromelain powders were produced following the process previously described, except for the dialysis step.

##### Modified Production of Bromelain Powders from Pineapple Peels

In short, one kilogram of pineapple peels was blended and homogenized with 50 mL of DIW (ratio: 20:1). Afterward, the mixture was filtered, dialyzed, and FD to obtain bromelain powders from pineapple peels (Figure 4).

#### 2.2.3. Phase III—Production of Bromelain Powders from Pineapple Stems and Basal Stems

**Figure 5:**
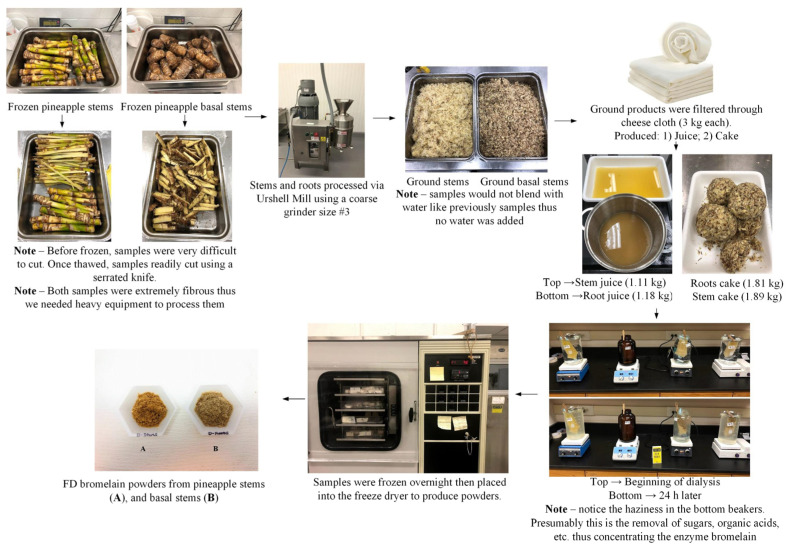
Production of freeze-dried (FD) bromelain powders from pineapple stems and basal stems.

The feasibility of producing FD bromelain powders from stems and basal stems was evaluated for comparison purposes and to determine if the proposed method could isolate bromelain with high enzymatic activity. Fresh stems and basal stems were grounded in an Urschel Mill using coarse grinder #3. Then, the ground products were filtered, dialyzed, and FD to produce FD bromelain powders from pineapple stems (Figure 5). The enzymatic activity and the crude protein of the resultant bromelain powders were measured.

### 2.3. Analyses

#### 2.3.1. Moisture Content, Total Soluble Solids (TSS), and pH of Pineapple By-Products

The moisture content was determined by AOAC Official Method 934.01 using a vacuum oven (Isotemp^®^ Model 281A, Thermo Fisher Scientific, Waltham, MA, USA) [19]. The cores, peels, and crowns were characterized for TSS and pH. The TSS were quantified using a portable digital handheld refractometer (Atago PAL-1, Cole-Palmer, Vernon Hills, IL, USA), while the pH was determined with a benchtop pH meter (ST2200-F, Ohaus, Parsippany, NJ, USA).

#### 2.3.2. Crude Protein Content Determination

The crude protein content was determined following the method reported by Nor et al. [1] using an automated nitrogen analyzer (Rapid N Exceed, Elementar, Langenselbold, Germany), which utilizes the Dumas combustion method. Dried samples (~0.5 g) were pelletized and placed in the autosampler of the nitrogen analyzer. Then, the nitrogen content was recorded. A conversion factor of 6.25 was used to determine the crude protein content, which was then converted to the wet basis (w.b.) using the moisture content. L-aspartic acid was used as the nitrogen calibration standard.

#### 2.3.3. Enzymatic Activity—Gelatin Digestion Method

The enzymatic activity of the bromelain powders was measured by following the Gelatin Digestion Unit (GDU) Analytical method of the Enzyme Development Corporation (see Appendix A).

##### Reagent Preparation

Five reagents, including 0.1 N NaOH and 0.1 N HCl solutions, were prepared to conduct the tests. The pH of the DIW was adjusted to 4.5 using the NaOH or HCl solutions, and the DIW with adjusted pH was used throughout the analysis. A gelatin substrate (GS) solution (pH 4.5) was prepared by dissolving 25 g of gelatin in 375 mL of hot DIW, then brought to a boil and cooled to 45 °C. Once cooled, the pH of the solutions was adjusted to 4.5 and brought to 500 mL with DIW. Afterward, the GS was placed in a 45 °C water bath, and a buffer solution (BS) with a pH of 4.5 was prepared by dissolving 15 g of NaCl in ~50 mL of DIW with continuous agitation. Next, 0.570 mL of acetic acid (1.049 g/mL at 25 °C) was added and the pH was adjusted to 4.5 with 0.1 N NaOH. A 3% HP solution with a pH of 4.5 was prepared by pipetting 2.5 mL of 30% HP into a 25 mL volumetric flask then diluted to volume with DIW. Finally, a 37% FA (pH 9.0) solution was prepared by pouring at least 100 mL of FA into a beaker, which was then adjusted to pH 9.0 with the 0.1 N NaOH solution.

##### Sample Testing

Enzyme-containing solutions (ESs) were prepared by placing ~0.500 g of bromelain powder into a 50 mL volumetric flask. Next, 8.3 mL of BS was added, and the mixture was left to stand for 30 min at room temperature. Afterward, the ES was brought to volume (50 mL) with the DIW and stirred for 15 min before testing. Then, two 100 mL beakers (1 for test, 1 for blank) were prepped by adding 25 mL of GS solution into each beaker; then, they were placed into the 45 °C water bath. After 5 min, 1.0 mL of the ES was added into the test beaker (beaker 1), and immediately, a timer was started for 20 min. Then, 0.1 mL of the 3% HP solution was added to the test solution, swirled, and incubated for exactly 5 min. Next, the test beaker was removed from the water bath, placed onto a stir plate, and after 10 s, the pH was recorded for the test solution (initial pH). Immediately following the pH recording, the pH was adjusted to 6.0 using the 0.1 N NaOH solution. Afterward, 10 mL of the 37% FA solution was added, and the pH was recorded at 10 s and 1 min. The test solution was then titrated to pH 9.0 with the 0.1 N NaOH solution and the titration volume of the NaOH used was recorded. This NaOH volume utilized was the test titer (T).

Once the timer for the test solution had 5 min left, 0.1 mL of 3% HP solution was added into the 100 mL beaker for the blank solution (beaker 2) and swirled. After 20 min of incubation in the 45 °C water bath, 1.0 mL of the ES was added, swirled, and incubated for 5 min. Next, the blank solution was removed from the water bath, placed onto the stir plate, and after 10 s, the pH of the test solution was recorded (initial pH). Then, the pH was adjusted to 6.0 with the 0.1 N NaOH solution. Next, 10 mL of the 37% FA solution (pH 9.0) was added and the pH reading was recorded at exactly 10 s and 1 min. The blank solution was then titrated to pH 9.0 with the 0.1 N NaOH solution and the titration volume of the NaOH used was recorded. The NaOH volume utilized is the blank titer (B). Note that each beaker was kept stirring on the stir plate, and the pH probe was left within the beaker for the duration of the test after being removed from the water bath.

##### Enzymatic Activity Calculation

Following triplicate readings for the prepared ES, the determination of the Gelatin Digesting Units (GDU) was performed. According to the Enzyme Development Corporation, 1 GDU is the amount of enzyme that liberates one mg of amino nitrogen from a standard gelatin solution after 20 min. of digestion at 45 °C and pH 4.5. The GDU/g was calculated following Equation (1):

(1) $$\frac{\mathrm{GDU}}{g}=\frac{\left(T-B\right)\times 14\times N\times 50}{\mathrm{Wt}.\left(g\right)}$$

where T = the test titer (mL of 0.1 N NaOH consumed during test titration); B = the blank titer (mL of 0.1 N NaOH consumed during blank titration); N = the normality of the NaOH utilized (0.1); and Wt. (g) = the initial weight of the bromelain powder.

#### 2.3.4. Enzymatic Activity—Casein Digestion

The enzymatic activity of bromelain powders was also measured by following the Casein Digestion Unit (CDU) Analytical method of the Enzyme Development Corporation with slight modifications (see Appendix B). This assay was split into three sections: (a) reagent preparation; (b) sample testing; and (c) enzymatic activity calculation.

##### Reagent Preparation

A casein substrate (CS) solution was prepared by placing a 4 L beaker on a stir/hot plate, filling it with 300 mL of DIW, and heating it to 50–60 °C. Meanwhile, in a 250 mL volumetric flask, 1.775 g of anhydrous disodium phosphate (Na_2_HPO_4_) was added and brought to volume with DIW. Then, 80 mL of the Na_2_HPO_4_ solution was added into a 150 mL beaker (substrate beaker) containing 0.6 g of casein and mixed until dissolved. The solution was then covered with aluminum foil and placed within the 4 L beaker water bath for 15 min with gentle stirring. Afterward, the substrate beaker was cooled to room temperature. Then, the pH of the CS solution was adjusted to 7.0 with a 0.1 N HCl solution. Next, the CS solution was transferred into a 100 mL volumetric flask, rinsed with DIW to remove excess particulate from the substrate beaker, and then brought to volume with DIW. Furthermore, a protein precipitant (TCA) solution was prepared by dissolving 9 g of trichloroacetic acid, 14.95 g of sodium acetate, and 9.9 g of acetic acid (1.049 g/mL at 25 °C) into a 500 mL volumetric flask and brought to volume with DIW. An enzyme diluent (EDTA) solution was prepared by dissolving 10.6 g of L-cysteine hydrochloride and 4.4 g of EDTA in 1.8 L of DIW in a 2 L volumetric flask. The solution was then adjusted to pH 4.5 with a 1 N NaOH solution and diluted to volume with DIW. Finally, a tyrosine standard solution was prepared by adding 50 μg/mL of L-Tyrosine in 0.1 N HCl.

##### Sample Testing

An enzyme solution was prepared by homogenizing 0.500 g of bromelain powder with a 100 mL EDTA solution. This mixture was further diluted B-fold, according to Equations (2) and (3), to the proper concentration of 40–50 CDU/mL. Note that this solution was tested within 30 min of preparation.

B = (Target CDU)/55(2)

where B = the dilution volumes (also called DF, used in Equation (4)); target CDU = the estimated bromelain activity (CDU/mg) that is expected and/or hypothesized.

(3) $$\frac{100}{500}\times \frac{100}{10}\times \frac{100}{B}=\mathrm{mL}\;\mathrm{in}\;\mathrm{final}\;\mathrm{dilution}$$

where B = the dilution volumes from Equation (2); this is the calculation for the pipetting volume that is added in the final dilution to the enzyme solution.

Once the enzyme solution was prepared, 5 mL of the CS solution was pipetted into three screw-cap test tubes, which were then incubated in a 37 °C water bath for 10 min. Next, 1 mL of the enzyme solution was added to test tube 1 (time zero), which was vortexed and immediately placed back into the water bath for 10 min. This step was repeated for test tubes 2 and 3. Afterward, 5 mL of the TCA solution was added to test tube 1, and then vortexed and returned back to the water bath. This latter step was repeated for test tube 2. After 10 min for test tube 3, 5 mL of the TCA solution and 1 mL of the original enzyme solution was added. Test tube 3 acted as the sample blank. Each test tube was incubated for an additional 30 min and then removed and allowed to cool to room temperature. Once cooled, the samples were then drawn up through a syringe (Sep-Pak Silica 20 cc Vac Cartridge, WAT036930, Waters, Milford, MA, USA) and then filtered through a syringe filter (0.45 μm PTFE membrane, Cat. No. 6785-2504, Whatman Inc., Clifton, NJ, USA) and placed into a quartz cuvette (CAT No. 14-958-128, Fisher Scientific, Hampton, NH, USA). The absorbance was read at 275 nm using a UV/Visible spectrophotometer (Model 7205, Jenway, London, UK) for the three test tubes and the tyrosine standard solution. Note that air was used to set the spectrophotometer to zero, and DIW was used to blank the spectrophotometer.

##### Enzymatic Activity Calculation

Following triplicate readings for the prepared enzyme solution, the determination of the CDU was performed. According to the Enzyme Development Corporation, a 1 CDU is defined as “the amount of enzyme that will liberate 1 μg of tyrosine after 1 min of digestion at 37 °C from a standard casein substrate solution at pH 7.0”. The CDU/mg was calculated following Equation (4) below:

(4) $$\frac{\mathrm{CDU}}{\mathrm{mg}}=\frac{\left(E_{t}-E_{b}\right)}{E_{s}}\times \frac{50}{1}\times \frac{11}{10}\times \mathrm{DF}$$

where E_t_ = the absorbance of the enzyme sample tube; E_b_ = the absorbance of the enzyme blank tube; E_s_ = the absorbance of the tyrosine standard; and DF = the dilution factor of the enzyme solution utilized in Section Sample Testing.

### 2.4. Statistical Analysis

Both experiments and analyses were carried out in triplicate. The mean and standard deviation (SD) values were calculated and reported. Then, a one-way analysis of variance (ANOVA) (α = 0.05) and Tukey’s studentized range test were carried out to determine differences among treatments at the significant level of *p* < 0.05. These tests were conducted using the RStudio statistical software version 2021.09.0 + 351 “Ghost Orchid” release for Windows (RStudio, Inc. Boston, MA, USA).

## 3. Results and Discussion

### 3.1. Phase I

#### 3.1.1. Enzymatic Activity of Bromelain Powders Extracted from Pineapple Cores, Peels, and Crowns

##### Cores

The pineapple cores had a moisture content of 92.2% (w.b.), a TTS of 9.1° Bx, and a pH of 3.54 (Table 1). The TTS results indicated the presence of soluble solids (mainly sugars and organic acids). According to De Ancos et al. [20], sucrose, glucose, and fructose are the main sugars, while citric, malic, and quinic acids are the main non-volatile organic acids found in pineapple pulp.

**Figure 6:**
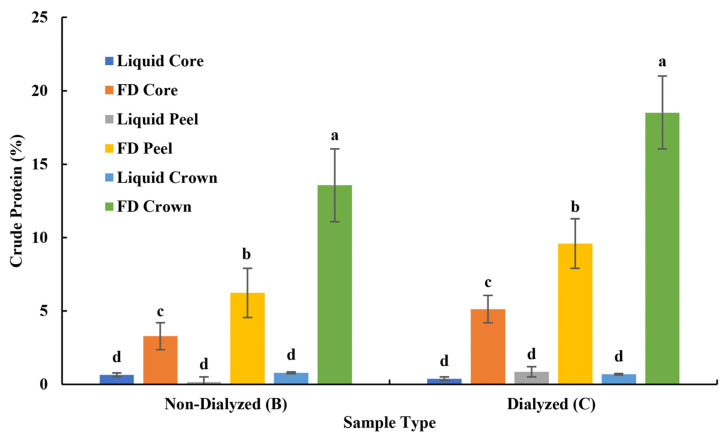
Crude protein (%, wet basis) of samples obtained during the bromelain extraction from pineapple by-products. ^a–d^ Means with different letters are significantly different (*p* < 0.05).

The pineapple cores were more manageable (compared to the other by-products) since they had a higher moisture content. In phase I, FD bromelain powders were successfully produced (Figure 2). The enzymatic activity measured with the gelatin and casein digestion methods of the resultant bromelain powders was 346 GDU/g and 115 CDU/mg, respectively. The crude protein concentration based on the nitrogen content was around 5% (Figure 6).

Other researchers have found similar bromelain activities in this pineapple portion, including 218 CDU/mg [8], 182 CDU/mL [21], and 152 CDU/mL [22]. In addition, Hebbar et al. [16] reported an enzymatic activity of 296 CDU/mg for bromelain obtained from pineapple cores using reverse micellar extraction and ultrafiltration. Gil and Maupoey [15] reported an enzymatic activity of 340 GDU/g for bromelain obtained from pineapple cores using a process that included solvent extraction, microfiltration, ultrafiltration, and freeze-drying.

Since our values are slightly lower than those previously reported, our team hypothesized that the enzymatic activity of these powders could be further explored and improved. Consequently, we explored alternative production methods in phase II.

##### Peels

The pineapple peels had a moisture content of 92.3% (w.b.), a TSS of 7.4° Bx, and a pH value of 3.72. The pineapple peels were more difficult to process than the cores, since this portion had more fibrous material, but were more manageable than the crowns (Figure 2). As in the previous case, FD bromelain powders were successfully developed from the peels. Interestingly, the enzymatic activity of the resultant FD bromelain powders was 92 GDU/g. Similarly, pineapple extracts from the pineapple variety Morris yielded 229 CDU/mL after extraction, and in fact, similar activities were obtained from the core and pineapple peels [21]. Our team decided to optimize the process in phase II. Ketnawa et al. [14] reported an enzymatic activity of 3–11 CDU/mg for bromelain extracted from pineapple peels using an organic solvent extraction process.

##### Crowns

The pineapple crowns had a pH value of 4.2 and a TSS of 1.9° Bx. These results are similar to previously reported values, where the crown pH value was 3.9 and the TSS was 1.6° Bx [23]. The degree brix values for these samples were lower compared to previous extractions. The samples were highly fibrous and difficult to process for further extraction. Bromelain powders were successfully produced with the same approach (Figure 3). However, the enzymatic activity of the resultant bromelain powders was negligible based on our enzymatic detection method (below the detection limits). According to Nadzirah et al. [23], the crown portion does have enzymatic activity, with approximately 500 CDU/mg after purification. This higher concentration of bromelain could be associated with small amounts of pulp during the extraction, since this portion had at least 2% pineapple pulp. Another group reported a significantly lower bromelain concentration (~150 CDU/mL) in crown extracts, the second lowest concentration after the stem [21]. Due to the significant variation among multiple studies, it is unclear whether the maturity stage of the pineapple, the time from harvesting to processing, or the extraction procedure affected the bromelain concentration/enzymatic activity.

#### 3.1.2. Crude Protein of Bromelain Powders

The preliminary results indicated that the bromelain powders from pineapple crowns had significantly (*p* < 0.05) higher crude protein than the powders made from peels and cores (Figure 4). As expected, the FD powders had significantly (*p* < 0.05) higher crude protein than the liquid samples before freeze-drying. Furthermore, dialysis significantly (*p* < 0.05) increased the crude protein content of the bromelain powders.

It was expected that the bromelain powders with a high crude protein content would have high enzymatic activity (due to the amino group on the amino acid chains). However, the bromelain powders obtained from the crowns did not show significant (*p* < 0.05) enzymatic activity but had a high crude protein content. This result may suggest that these powders contained other nitrogen-based products, such as chlorophyll; therefore, the quantification of crude protein may have been overestimated. It has been previously reported that the protein content in pineapple is around 1% [21,23]. Some researchers have described that the yield of bromelain from pineapple crowns can be as low as 0.25% after purification steps [24]. The crowns were not used in phase II of this study due to the low enzymatic activity of their bromelain powders.

### 3.2. Phase II—Improved Bromelain Extraction

The previous results from phase I indicated that the bromelain powders from cores and peels had promising enzymatic activities. Therefore, the original approach used in phase I was modified to obtain bromelain powders with potentially higher enzymatic activity. Fresh pineapple cores were processed similarly to those in phase I, but this time without pH modification (Figure 4). Alternatively, a second modification was evaluated, including the bromelain extraction without the pH modification and ultrafiltration steps. The results of both modifications were assessed to observe the effect of each step.

#### 3.2.1. Bromelain Powders from Pineapple Cores without pH Adjustment

Our team hypothesized that increasing the pH of the liquid mixture to seven during the extraction process (as in phase I) reduced the amount of bromelain collected. Hence, the pH adjustment was removed from the process. The results revealed that the consequent non-pH-adjusted bromelain powders had an enzymatic activity of 694 GDU/g and 124 CDU/mg, which is significantly (*p* < 0.05) greater than the enzymatic activity of the bromelain powders from cores developed in phase I (Table 2). Seven grams of bromelain powder was produced from 1 kg of fresh cores (0.7% yield) (Table 3).

It has been reported that bromelain from pineapple cores is an acidic enzyme, and its pI is 4.6; therefore, this could be the reason why higher concentrations of protein were obtained using this procedure [11]. These results are similar to those results obtained by Gil and Maupoey [15], where lyophilized powder had an enzymatic activity between 340 and 805 GDU/g. It is essential to highlight that fruit bromelain usually has less enzymatic activity than stem bromelain [25]. To make a fair comparison, the enzymatic activity of a commercial bromelain dietary supplement was evaluated (Bromelain, Best Naturals, Kenilworth, NJ, USA). The supplement claimed an enzymatic activity of 600 GDU/g; however, our team only detected 424 GDU/g and 65.6 CDU/mg in this commercial product. These results suggest that the bromelain powders obtained from pineapple cores had more enzymatic activity than commercially available bromelain supplements. Our finding also creates questions about the shelf stability of bromelain powders, since shelf stability may be a problem for bromelain powders used in different applications. Therefore, further evaluation is needed to ensure the proper shelf-life of this product.

Since the first results from phase II were encouraging, the production of bromelain powders was simplified to see the quality of bromelain obtained. Therefore, bromelain powders were produced without the pH adjustment and ultrafiltration steps. As a result, approximately 57 g of FD bromelain powder was made from 1 kg of fresh cores (5.7% yield), and a significantly (*p* < 0.05) higher yield rate was obtained compared to the production of bromelain powders using ultrafiltration. However, the resultant bromelain powders had a significant (*p* < 0.05) lower enzymatic activity (204 GDU/g and 59 CDU/mg) compared to the powders produced with ultrafiltration (Table 3). These results suggest that ultrafiltration is necessary to make bromelain powders with high enzymatic activity. This type of bromelain powder may be used in meat tenderizer applications that do not require enzymes with a high enzymatic activity [26].

#### 3.2.2. Bromelain Powders from Pineapple Peels without pH Adjustment

The resultant bromelain powders from peels without the pH adjustment had an enzymatic activity of 1179 GDU/g and 217 CDU/mg (Table 3). This was significantly (*p* <0.05) higher than the enzymatic activity of bromelain powders from cores. Approximately six grams of bromelain powder was produced from 1 kg of fresh peels (0.6% yield). In addition, our team evaluated the enzymatic activity of the commercial bromelain standard enzyme obtained from stems (Cat. No. 402830250, Thermo Fisher Scientific, Waltham, MA, USA). The standard was analyzed within 48 h of receipt. The product claimed an enzymatic activity of 1200 GDU/g (Table 3). However, our team only detected 553 GDU/g and 229 CDU/mg of enzymatic activity. It is believed that the lower enzymatic activity (compared to the claimed activity) of the bromelain standard may be due to the reduction in enzymatic activity during storage. Regardless of the difference between the bromelain standard and the actual activity found by our team, the isolated bromelain produced by the proposed approach had a higher enzymatic activity.

### 3.3. Phase III—Production of Bromelain Powders from Pineapple Stems and Basal Stems

Contradictory information regarding the prevalence of bromelain in pineapple stems has been found in the scientific literature. Previous research has demonstrated that bromelain can be found in pineapple stems; however, their activity seems equivalent or lower than bromelain obtained from other parts of the fruits [21]. Other researchers have suggested that stem bromelain’s enzymatic activity is higher than fruit bromelain’s. Stem bromelain has a molecular weight of 27 kDa, while fruit bromelain has molecular weights between 19 and 23 kDa [27,28]. According to Chakraborty et al. [27], multiple proteinase fractions have different molecular weights in each plant portion with slight changes in the amino acid terminals. Therefore, this complex mixture of proteinases makes it extremely complicated to compare among studies, since their multiple methods of extraction, multiple portions of the stem, and the maturity of the plants, among other factors, may have influenced the enzymatic activity of the resultant bromelain-based products.

In general, the stem of the pineapple plant is one of the most significant by-products at the farm level, with fewer upcycling opportunities. Therefore, there is enormous interest in evaluating this byproduct as a raw material for producing high-value products such as bromelain. For example, some commercially available dietary supplements use pineapple stems to produce bromelain-based products. Therefore, our research explored the idea of obtaining bromelain powders from stems and the basal part of the stem, which is believed to have a higher bromelain concentration (Figure 5). Frozen stems were thawed and ground in an Urschel Mill. Then, the ground stems were pressed, and the resultant liquid was dialyzed and FD. The enzymatic activity of FD bromelain from stems (upper portion) was 23 GDU/g and 54 CDU/mg. Approximately 9.6 g of powder was obtained from 3 kg of fresh stems (0.3% yield) (Table 4). Furthermore, the crude protein content of the bromelain powders was 10.5% (*w*/*w*). The resulting bromelain powders had a lower enzymatic activity (compared to the bromelain powders obtained from the peels and cores).

On the other hand, basal stems were also processed similarly to pineapple stems (Figure 5). Interestingly, the resultant bromelain powders from basal stems demonstrated a very high enzymatic activity. Furthermore, the enzymatic activity of the resultant bromelain powders was 2909 GDU/g and 717 CDU/mg. The bromelain powders from basal stems showed the highest enzymatic activity of all powders developed and tested in this study. Approximately 20.4 g of powder was produced from 3 kg of fresh basal stems (0.7% yield). In addition, the crude protein content of the bromelain powders was 27.7% (*w*/*w*). According to Rowan [29], stem bromelain (an endopeptidase) is a glycosylated, single-chain protein of 24.5 kDa, with a pI of 9.55, containing seven cysteines. The secondary structure of the enzyme is relatively stable at pH values between 7 and 10. It is possible that this enzyme naturally protects plants from parasites, pathogens, and animals. Rowan [29] suggested that 90% of the proteolytic activity of stem extracts is due to stem bromelain. These findings agree with those previously reported, where stem bromelain has better enzymatic activity than fruit bromelain [25]. Misran et al. [30] reported similar findings where the stems had low enzymatic activity but did not mention the basal stem enzymatic activity. Certainly, basal stems are portions of the pineapple that are not fully explored and could be an option as a by-product containing bromelain from farming operations. Additionally, further exploration is needed to understand basal stems’ enzymatic activity better and determine the shelf-life stability of commercial applications of bromelain powders obtained from pineapple basal stems.

## 4. Conclusions

Bromelain powders with promising enzymatic activities were successfully produced from pineapple by-products (cores, peels, stems, and basal stems) via organic solvent-free extraction, ultrafiltration, and freeze-drying. This study was divided into three phases. In phase I, the feasibility of producing bromelain powders from pineapple cores, peels, and crowns was evaluated. Bromelain powders from pineapple crowns showed no enzymatic activity; hence, the crowns were removed from the rest of this study, while bromelain powders from cores and peels showed promising results. In phase II, the production of bromelain powders with high enzymatic activity was further optimized. Finally, in phase III, the feasibility of producing bromelain powders from pineapple stems and basal stems was evaluated using the optimized methods developed in phase II. Once the production process was improved, bromelain powders produced from basal stems had the highest enzymatic activity (2909 GDU/g and 717 CDU/mg) followed by those produced from peels (1179 GDU/g and 217 CDU/mg) and cores (694 GDU/g and 124 CDU/mg), respectively. This study confirms previous knowledge regarding a higher enzymatic activity of stem bromelain than fruit bromelain and presents a more sustainable approach to producing bromelain powders. Future studies should evaluate the shelf-life stability of bromelain powders in different conditions. The resultant powders have potential applications in food, animal feed, and dietary supplements.

## Figures and Tables

**Table 1:** Moisture, total soluble solids (TSS), and pH of pineapple cores, crowns, and peels.

	Moisture (g/100 g, Wet Basis)	TSS (° Bx)	pH
Cores	92.2 ± 0.4 ^a^	9.1 ± 0.1 ^a^	3.54 ^c^
Peels	92.3 ± 0.4 ^a^	7.4 ± 0.1 ^b^	3.72 ^b^
Crowns	18.7 ± 0.3 ^b^	1.9 ± 0.1 ^c^	4.24 ^a^

^a–c^ Means with different letters within the same column are significantly different (*p* < 0.05).

**Table 2:** Enzymatic activity of bromelain powders produced from pineapple crowns, cores, and peels.

Treatment	Enzymatic Activity	
	Gelatin Digestion (GDU/g)	Casein Digestion (CDU/mg)
Cores	346 ^a^	115
Peels	92 ^a^	N.A.
Crowns	N.D.	N.D.

N.A. = Could not be determined. N.D. = Not detected. ^a^ Means with different letters within the same column are significantly different (*p* < 0.05).

**Table 3:** Yield and enzymatic activity of bromelain powders obtained from phase II.

No.	Treatment		Enzymatic Activity	
		Yield (%)	Gelatin Digestion (GDU/g)	Casein Digestion (CDU/mg)
1	Cores, no pH adjustment, non-dialyzed	5.7	204 ^b^	59 ^c^
2	Cores, no pH adjustment	0.7	694 ^b^	124 ^b^
3	Peels, no pH adjustment	0.6	1179 ^a^	217 ^a^
4	Commercial bromelain tablets (Control 1)	N.A.	424 ^b^	66 ^c^
5	Bromelain standard (1200 GDU/g) (Control 2)	N.A.	553 ^b^	229 ^a^

N.A.—could not be determined. Values are means ± standard deviation of triplicate determinations for gelatin digestion (GDU/g). Values are means ± standard deviation of duplicate determinations for casein digestion (CDU/mg). ^a–c^ Means with different letters within the same column are significantly different (*p* < 0.05).

**Table 4:** Yield and enzymatic activity of bromelain powders obtained from phase III.

No.	Treatment		Enzymatic Activity		Crude Protein (%)
		Yield (%)	Gelatin Digestion (GDU/g)	Casein Digestion (CDU/mg)	
1	Stems	0.3	23 ^b^	54 ^b^	10.5 ^b^
2	Basal stems	0.7	2909 ^a^	717 ^a^	27.7 ^a^

Values are means of triplicate determinations for gelatin digestion (GDU/g). Values are means of duplicate determinations for casein digestion (CDU/mg). ^a–c^ Means with different letters within the same column are significantly different (*p* < 0.05).

**Figure A1:**
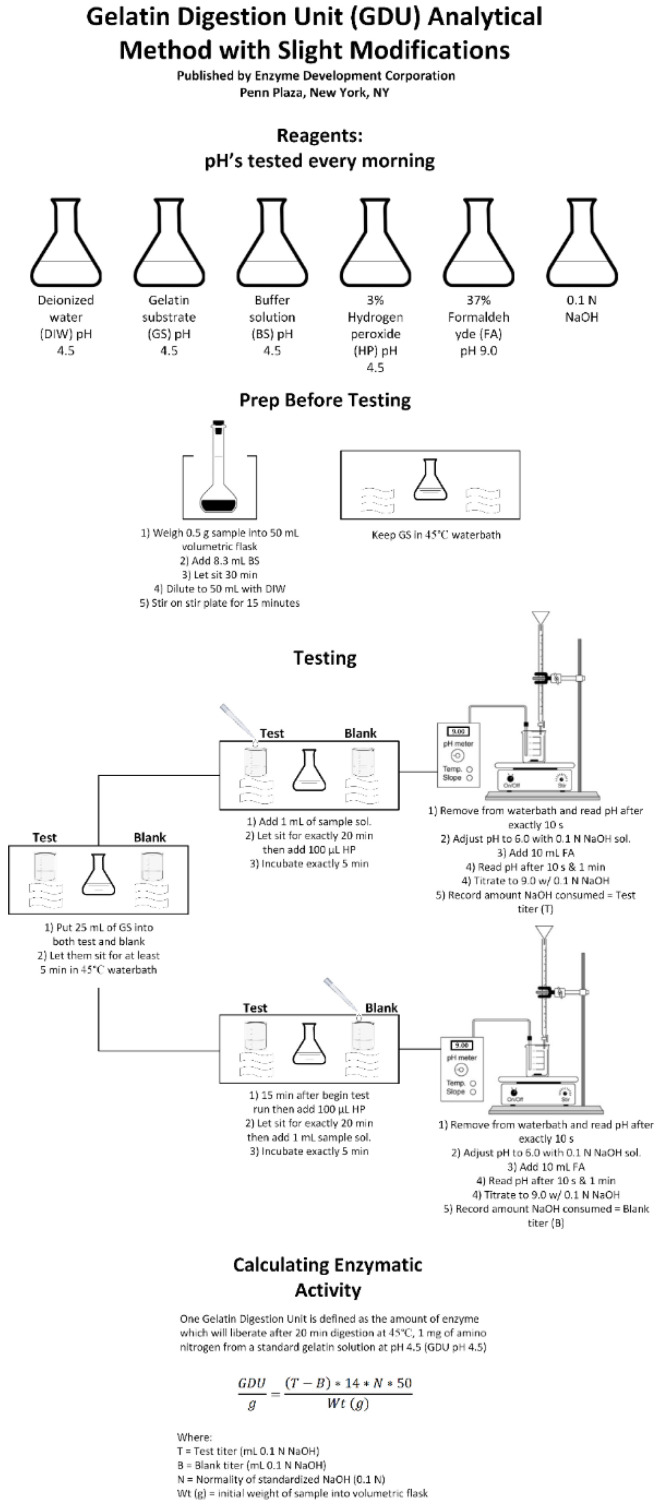
General overview of the gelatin digestion method to determine enzymatic activity of bromelain powders.

**Figure A2:**
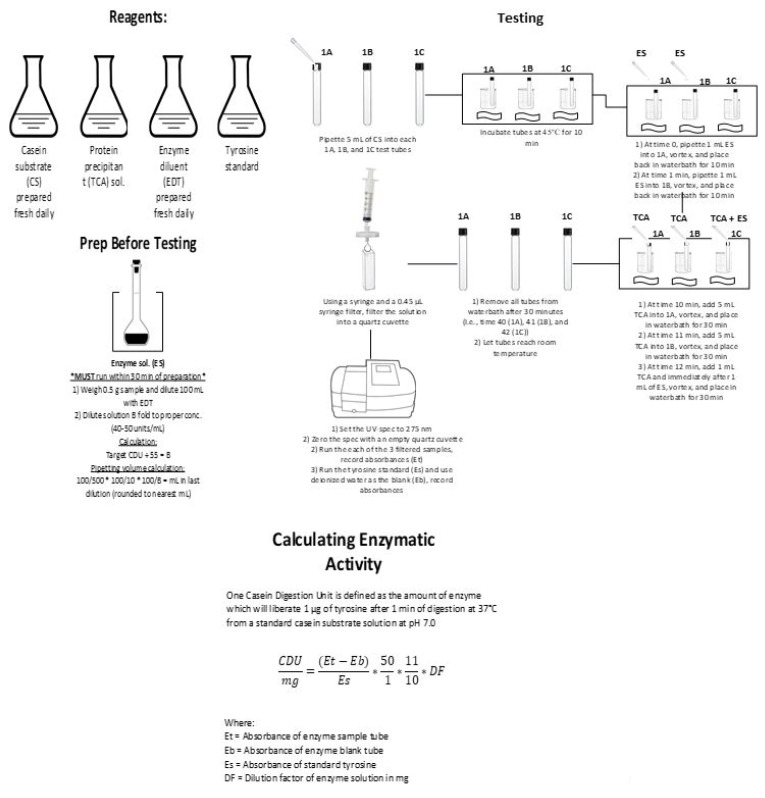
General overview of the casein digestion method to determine enzymatic activity of bromelain powders.

## Data Availability

Data is contained within the article.
